# Characteristics of coronary artery disease in symptomatic type 2 diabetic patients: evaluation with CT angiography

**DOI:** 10.1186/1475-2840-9-74

**Published:** 2010-11-10

**Authors:** Zhi-gang Chu, Zhi-gang Yang, Zhi-hui Dong, Zhi-yu Zhu, Li-qing Peng, Heng Shao, Ci He, Wen Deng, Si-shi Tang, Jing Chen

**Affiliations:** 1 Department of Radiology, West China Hospital, Sichuan University, Chengdu, Sichuan 610041, PR China; 2 State Key Laboratory of Biotherapy, West China Hospital, Sichuan University, Chengdu, Sichuan 610041, PR China; 3 Department of Pathology, West China College of Stomatology, Sichuan University, Sichuan 610041, PR China

## Abstract

**Background:**

Coronary artery disease (CAD) is a common and severe complication of type 2 diabetes mellitus (DM). The aim of this study is to identify the features of CAD in diabetic patients using coronary CT angiography (CTA).

**Methods:**

From 1 July 2009 to 20 March 2010, 113 consecutive patients (70 men, 43 women; mean age, 68 ± 10 years) with type 2 DM were found to have coronary plaques on coronary CTA. Their CTA data were reviewed, and extent, distribution and types of plaques and luminal narrowing were evaluated and compared between different sexes.

**Results:**

In total, 287 coronary vessels (2.5 ± 1.1 per patient) and 470 segments (4.2 ± 2.8 per patient) were found to have plaques, respectively. Multi-vessel disease was more common than single vessel disease (*p *< 0.001), and the left anterior descending (LAD) artery (35.8%) and its proximal segment (19.1%) were most frequently involved (all *p *< 0.001). Calcified plaques (48.8%) were the most common type (*p *< 0.001) followed by mixed plaques (38.1%). Regarding the different degrees of stenosis, mild narrowing (36.9%) was most common (*p *< 0.001); however, a significant difference was not observed between non-obstructive and obstructive stenosis (50.4% vs. 49.6%, *p *= 0.855). Extent of CAD, types of plaques and luminal narrowing were not significantly different between male and female diabetic patients.

**Conclusions:**

Coronary CTA depicted a high plaque burden in patients with type 2 DM. Plaques, which were mainly calcified, were more frequently detected in the proximal segment of the LAD artery, and increased attention should be paid to the significant prevalence of obstructive stenosis. In addition, DM reduced the sex differential in CT findings of CAD.

## Background

Diabetes mellitus (DM) is a disorder of carbohydrate, protein and fat metabolism. Chronic hyperglycemia in DM damages various organs and leads to a series of complications. Blood vessels are the commonly affected targets and relevant complications are the leading causes of death in patients with type 2 DM [1]. Among cardiovascular complications, coronary artery disease (CAD) has been observed most frequently, and it imposes a huge health burden in all countries [2,3]. The severity of CAD in diabetic patients may be determined by its characteristics associated with DM. Therefore, it is necessary to study the manifestations of CAD in diabetic patients to comprehensively understand this complication.

Coronary angiography is regarded as the golden standard for evaluation of coronary artery stenosis, but this method could not depict the type of plaque and had some risks during manipulation. Magnetic resonance angiography enabled the assessment of plaque composition and may have reflected the real culprit, but it could not be widely used in the near future because of high costs and complex methodology [4]. However, multi-detector row CT (MDCT), especially dual-source CT (DSCT), could accurately determine plaque composition and assess the degrees of luminal narrowing [5-8]. The purpose of this study is to determine the characteristics of CAD in diabetic patients by using DSCT, thus increasing the understanding of the severity of this complication.

## Methods

### Study population

From 1 July 2009 to 20 March 2010, 138 patients with type 2 DM underwent coronary DSCT angiography (DSCTA) examination because of chest pain (66%), shortness of breath (23%), palpitation (10%) and syncope (1%). Patients with coronary artery plaques and those who had complete clinical data and laboratory results were included in this study. A total of 133 (96.4%) patients met the above-mentioned criteria except five (3.6%) who had no plaques but had myocardial bridges. The exclusion criteria were poor CT scan quality that could not be used for analysis (6 cases) and a history of CAD, stenting or bypass (14 cases). Finally, 113 (113/138, 81.9%) patients were enrolled in this study. The clinical data of patients included age, sex, height and weight, DM history, smoking history, hypertension, use of medications and other complications related to DM. Laboratory results included fasting blood glucose, glycosylated haemoglobin, triglyceride, cholesterol, low-density lipoprotein cholesterol and high-density lipoprotein-cholesterol.

### CT protocols

Coronary CT angiography (CTA) was performed using a Siemens DSCT scanner (SOMATOM Definition, Siemens Medical Solutions, Forchheim, Germany). Beta-blocker preparation was not used for reducing the heart rate. The scanning scope was from the tracheal bifurcation to 20 mm below the inferior cardiac apex. A 70-90-mL (dependent on body mass index) bolus of iodinated contrast agent (iopamidol, 370 mg of iodine/mL; Bracco Sine Pharmaceutical Corp. Ltd, Shanghai, China) was injected into the antecubital vein at a flow rate of 5 mL/sec. Next, a 20-mL saline chaser was injected at the same rate. Scan parameters were tube voltage 100-120 kV (adapted to body mass index); tube current, 220 mAs; collimation, 64 × 0.6 mm; rotation time, 0.33 s and pitch, 0.2-0.5 (adapted to the heart rate). Retrospective electrocardiographic gating was used to eliminate cardiac motion artefacts. Data acquisition was completed within 8-10 s.

### Image analysis

An initial data set was reconstructed and a group of images with optimal quality was transferred to a post-processing workstation (Syngo-Imaging, Siemens Medical Solution Systems, Forchheim, Germany) for image analysis. Alternative image reconstruction methods for evaluation of coronary artery plaques included maximum intensity projection, multiplanar reconstruction, curvature plane reconstruction and volume reconstruction.

**Figure 1:**
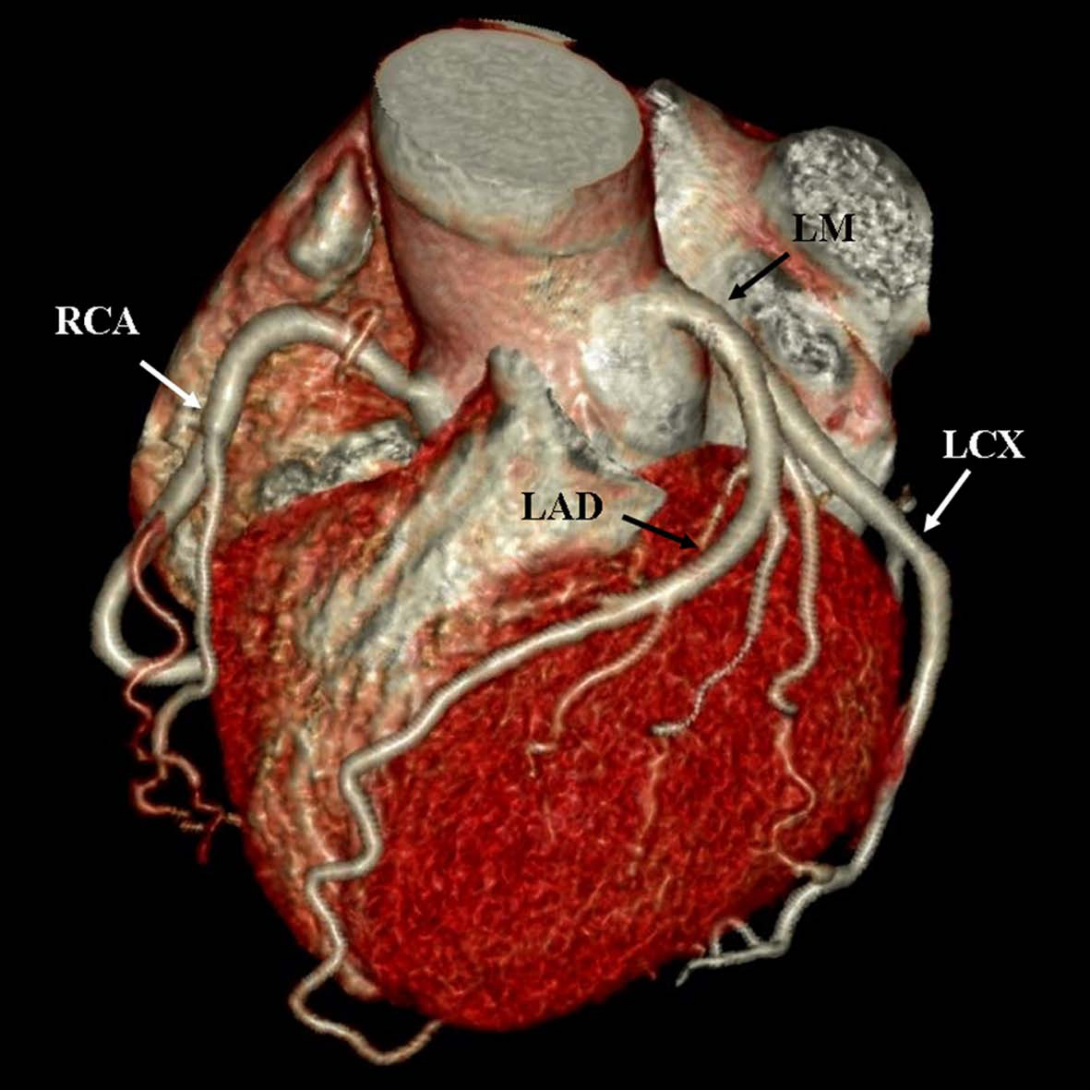
**Volume rendering image shows the branches of coronary artery**. LM: left main; LAD: left anterior descending; LCX: left circumflex; RCA: right coronary artery.

**Figure 2:**
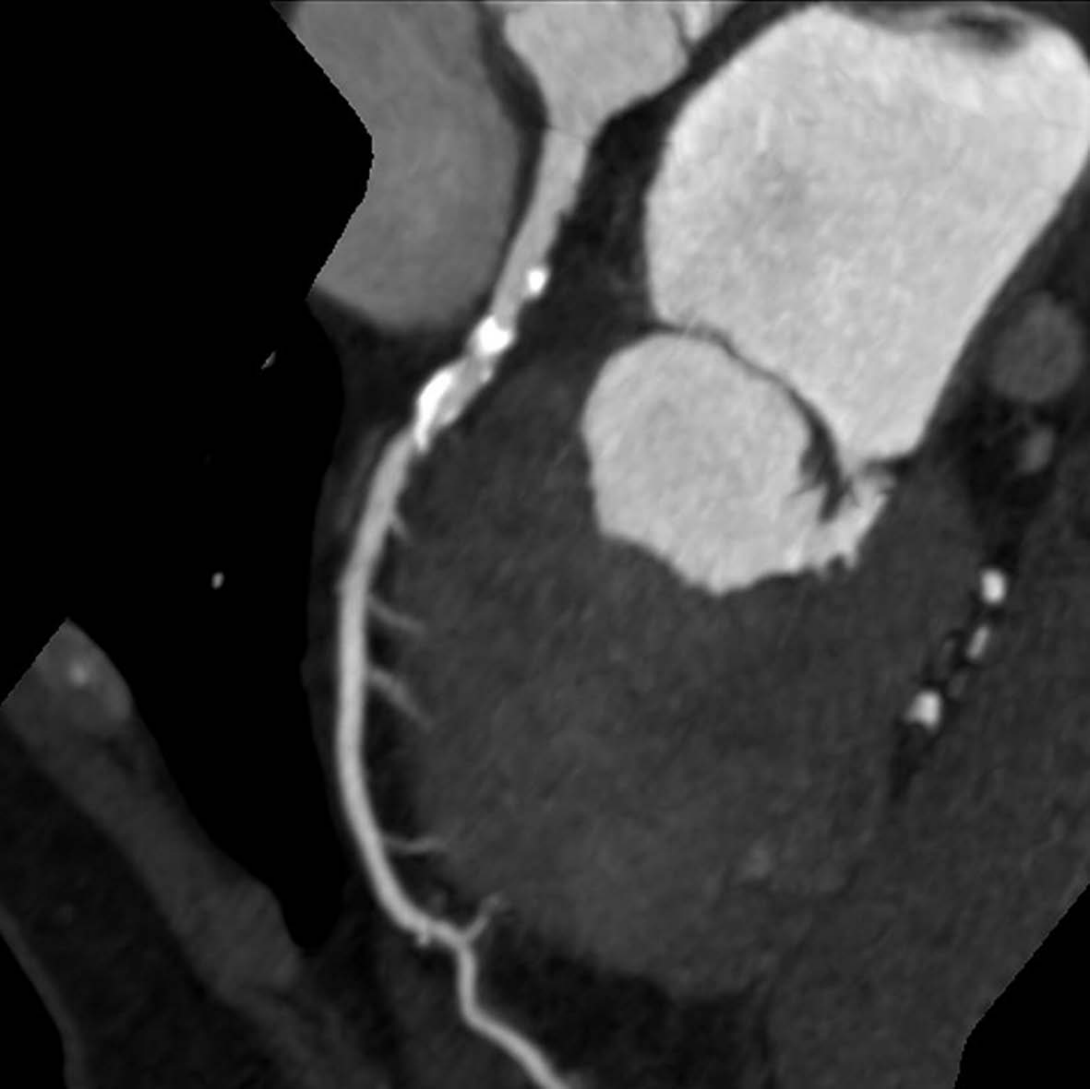
**This image shows many calcified plaques, the density of which is significantly higher than contrast-enhanced lumen**.

**Figure 3:**
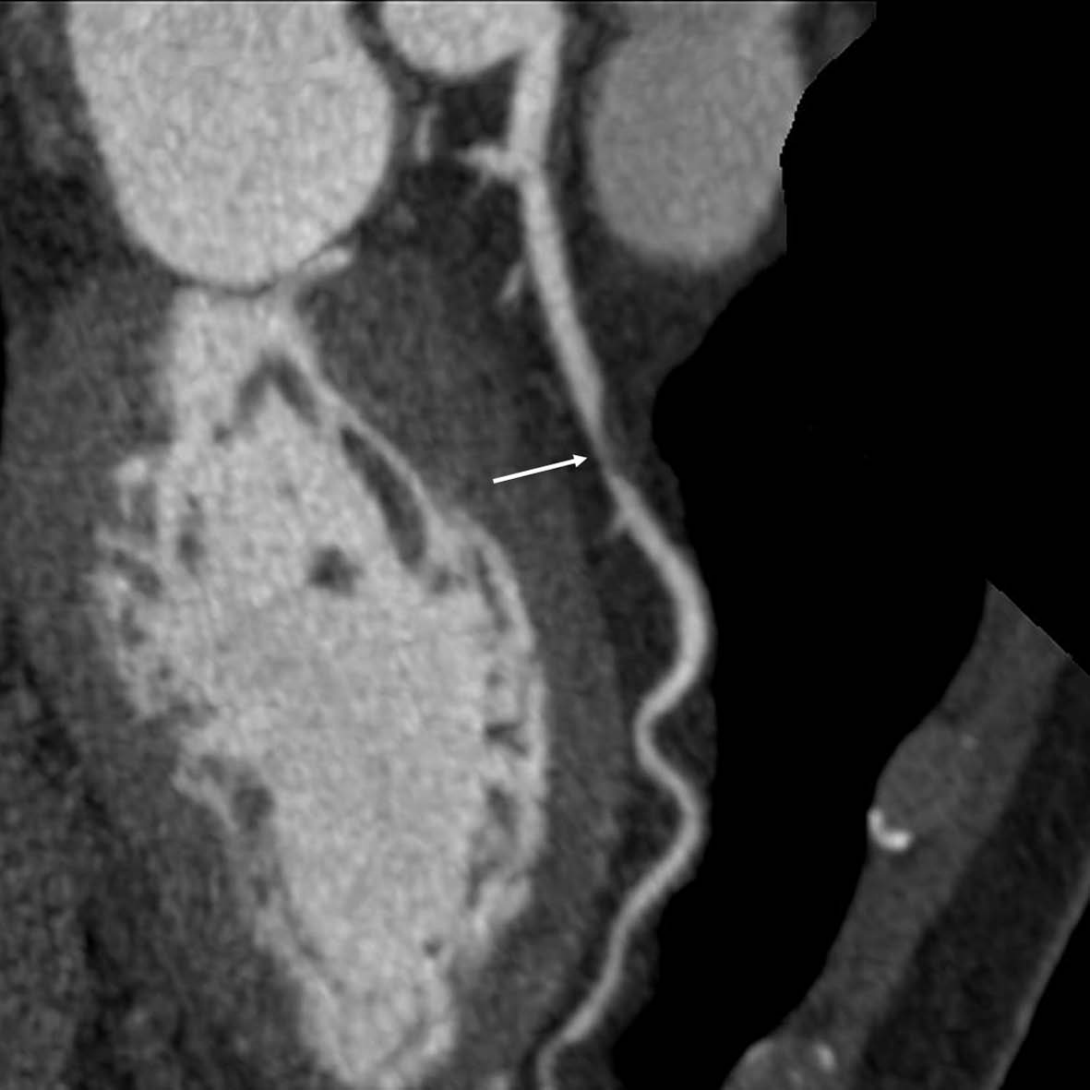
**This image shows a non-calcified plaque, which manifested as an eccentric filling defect and causes significant stenosis (arrow)**.

**Figure 4:**
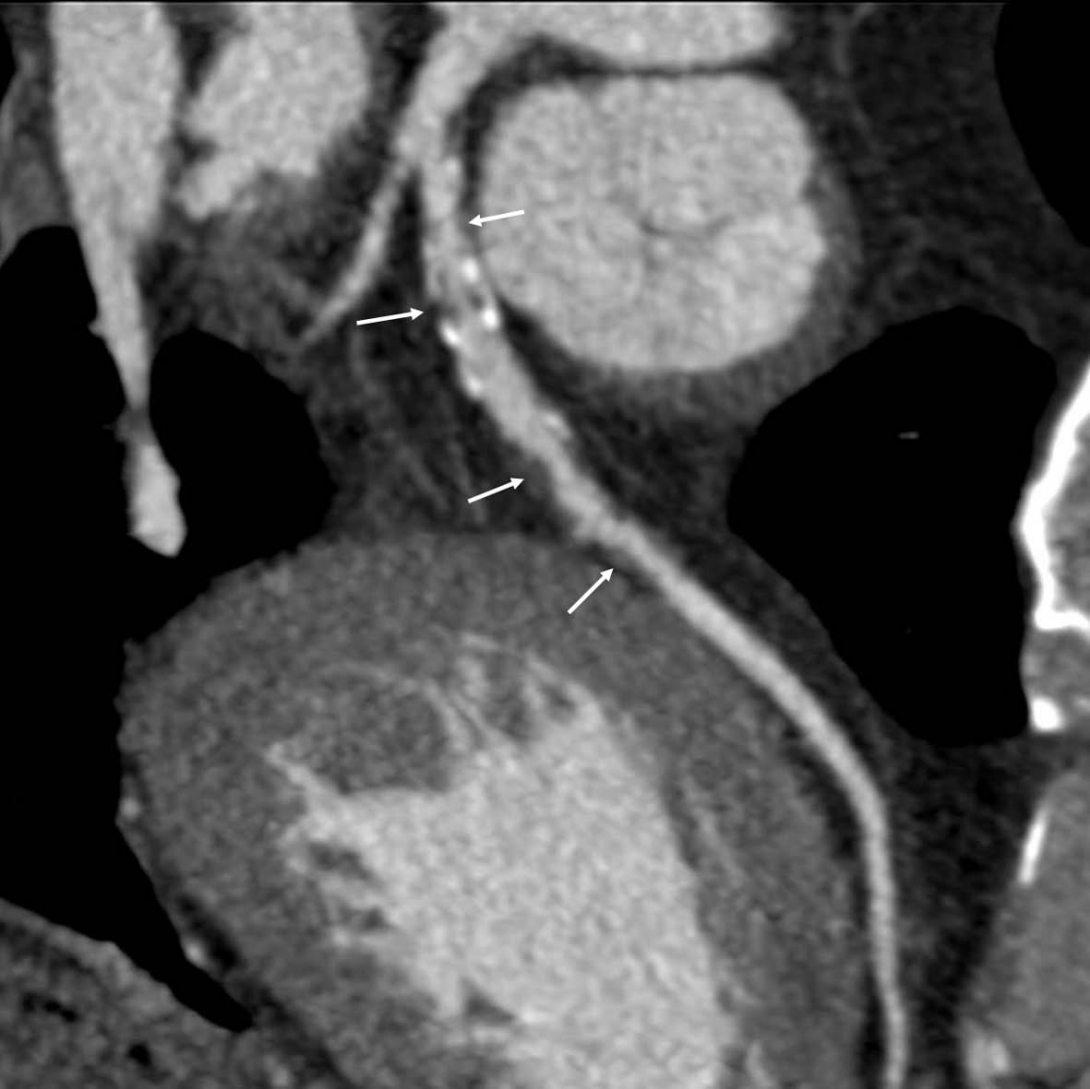
**This image shows many mixed plaques, which manifested as many calcified plaques and non-calcified plaques (arrows) mix together**.

Two cardiovascular radiologists independently analyzed the images. Discrepancies in their interpretations were resolved by consensus. Both observers were blinded to the medical histories, clinical diagnoses and results of other investigations for all patients. Number of diseased coronary vessels and segments, number and types of plaques and grading of stenosis caused by plaques were evaluated. In this study, coronary arteries were divided into four branches: left main (LM), left anterior descending (LAD), left circumflex (LCX) and right coronary artery (RCA) (Figure 1). According to the standard of the American Heart Association, the left and right coronary arteries were divided into 15 segments [9]. Plaques were classified as calcified plaque (plaques with higher CT density than contrast-enhanced lumen) (Figure 2); non-calcified plaque (plaques with lower CT attenuation than contrast-enhanced lumen without any calcification) (Figure 3) and mixed plaque (non-calcified and calcified elements in single plaque) (Figure 4) [10]. Overall, coronary artery stenosis caused by plaques was classified as obstructive and non-obstructive using a 50% threshold of luminal narrowing. In addition, grading of stenosis was further classified as normal appearing (<25%), mild (25%-49%), moderate (50%-74%) and severe (≥75%) narrowing [11]. The degree of stenosis was assessed on the basis of two orthogonal views.

### Statistical analysis

Clinical data, laboratory results, number of diseased coronary vessels and segments, as well as number and types of plaques and grading of luminal narrowing were analyzed statistically in each patient. Continuous variables were expressed as mean ± standard deviation and categorical variables as number and percentage. The chi-square test was used to compare the difference between multi- and single-vessel diseases or plaque distributions among different vessels and segments as well as the different types of plaques and degree of stenosis. Independent sample *t*-test was used to compare the manifestations of CAD between male and female diabetic patients. The *κ *statistic was used to calculate the interobserver variability. Statistical analysis was performed using the SPSS statistical package (version 13.0 for Windows, SPSS Inc., Chicago, Illinois, USA). Two-tailed *p *value of less than 0.05 was considered statistically significant.

## Results

The 113 patients who met the criteria had good image quality, and their coronary CTA was used to analyze plaque composition and assess grading of stenosis. The mean radiation dose from CTA examination per patient was 4.9 ± 1.7 mSv (range, 1.8-8.9 mSv). There was perfect agreement between the two observers on the type of plaques (*κ *= 0.92) and grading of stenosis (*κ *= 0.90) observed on CT scan.

### Clinical and laboratory characteristics of the patient population

The baseline clinical data and laboratory results are summarized in Table 1. Blood glucose level was controlled with oral hypoglycemic agents (e.g. Repaglinide, Acarbose, Glibenclamide and Metformin) in 78 (69.0%) patients and with insulin in 21 (18.6%) patients. Fourteen patients (12.4%) did not use any hypoglycemic agents but had adjusted their diets or their diabetic status was discovered for the first time in this study.

**Table 1:** Baseline clinical and laboratory characteristics of the 113 patients

Characteristic	Value
Age (years)	68 ± 10 (41-86)
Men/women	70/43
Body mass index (kg/m^2^)	25.4 ± 3.0 (7.3-34.1)
Duration of diabetes mellitus (years)	8.6 ± 6.2 (0-30)
Current Smoking	41(36)
Hypertension	81(72)
Diabetic nephropathy	21 (18.6)
Diabetic retinopathy	67 (59.3)
Diabetic foot	16 (14.1)
Diabetic ketoacidosis	6 (5.3)
Other DM-related complication	27 (23.9)
Ejection fraction of left ventricle (%)	65 ± 9 (21-80)
Fasting blood-glucose (mmol/L)	7.9 ± 3.3 (3.8-19.0)
Glycosylated hemoglobin level (%)	7.6 ± 2.1 (5.3-15.0)
Triglyceride (mmol/L)	2.0 ± 1.4 (0.5-7.8)
Cholesterol (mmol/L)	4.2 ± 1.1 (1.9-7.8)
HDL-C (mmol/L)	1.2 ± 0.3 (0.5-2.1)
LDL-C (mmol/L)	2.4 ± 1.0 (0.6-5.0)

Data are expressed as means ± SD (range) or n (%). Normal range (Fasting blood-glucose: 3.9-5.9 mmol/L; Glycosylated hemoglobin: 4.5-6.1%; Triglyceride: 0.29-1.83 mmol/L; Cholesterol: 2.8-5.7 mmol/L; HDL-C: >0.9 mmol/L; LDL-C: <4.0 mmol/L). HDL-C: high density lipoprotein cholesterol; LDL-C: low density lipoprotein cholesterol.

### Extent and anatomic distribution of coronary artery plaques

**Figure 5:**
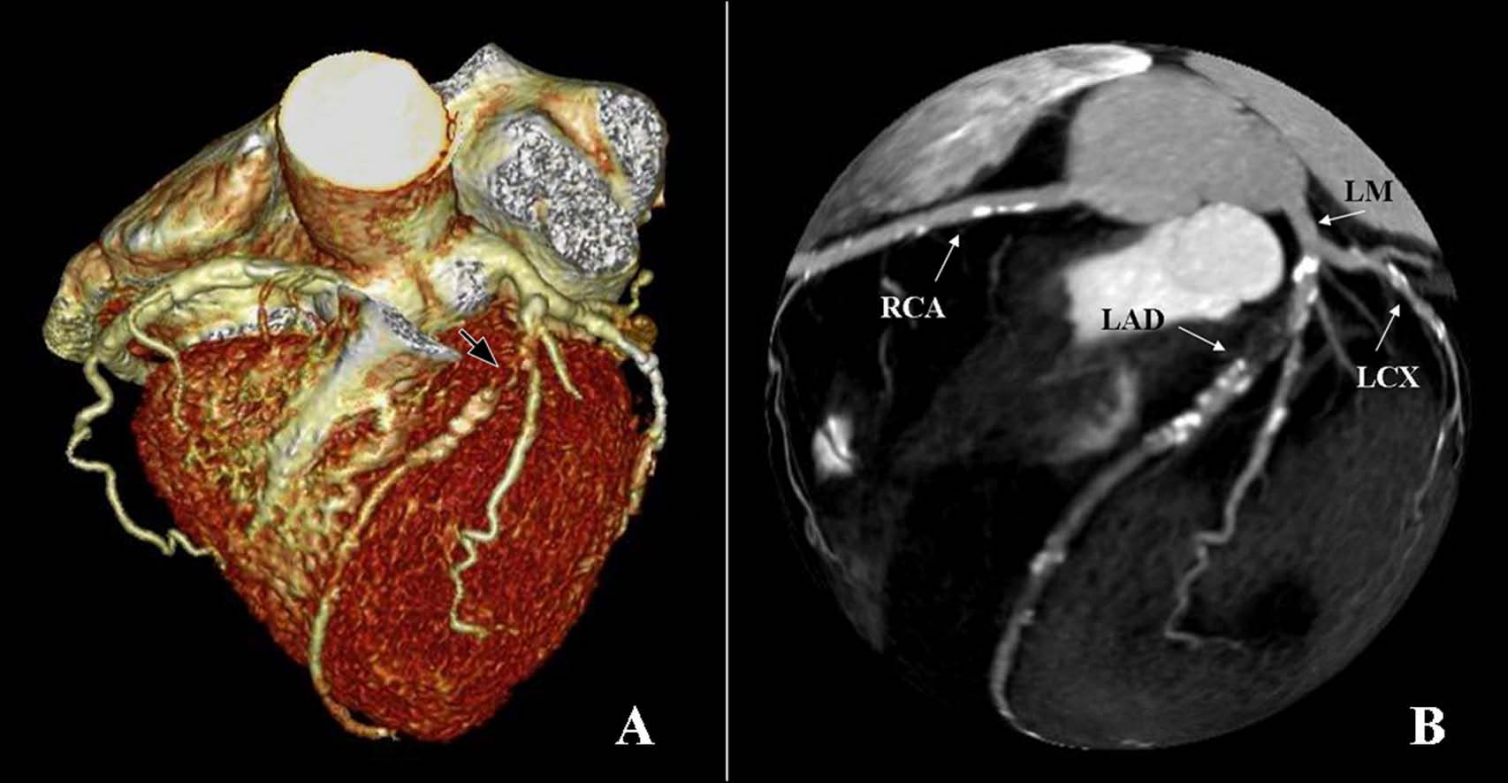
**A male diabetic patient with multi-vessel disease**. **A**, Volume rendering image shows the edge of left and right coronary vessels is unsmooth, and the proximal segment of LAD artery has significant stenosis (arrow). **B**, Globe image of coronary arteries shows there are many plaques distributing in the whole course of the LAD, LCX and RAC arteries.

A total of 287 coronary vessels (2.5 ± 1.1 per patient; range, 1-4) and 470 segments (4.2 ± 2.8 per patient; range, 1-13) were found to have plaques. Number of patients with multi-vessel (≥2) disease (Figure 5) was significantly higher than that with single-vessel disease [85 of 113 (75.2%) vs. 28 of 113 (24.8%), *p *< 0.001]. The most common diseased coronary vessel was the LAD artery [35.9% (LAD) vs. 27.2% (RCA), 22.6% (LCX) or 14.3% (LM); all *p *< 0.001). Furthermore, its proximal segment was the most common diseased coronary segment (90/470, 19.1%, *p *< 0.001), followed by the proximal segment of RCA (63/470, 13.4%) and the middle segment of the LAD artery (62/470, 13.2%).

### Types of coronary artery plaque and coronary artery stenosis

**Figure 6:**
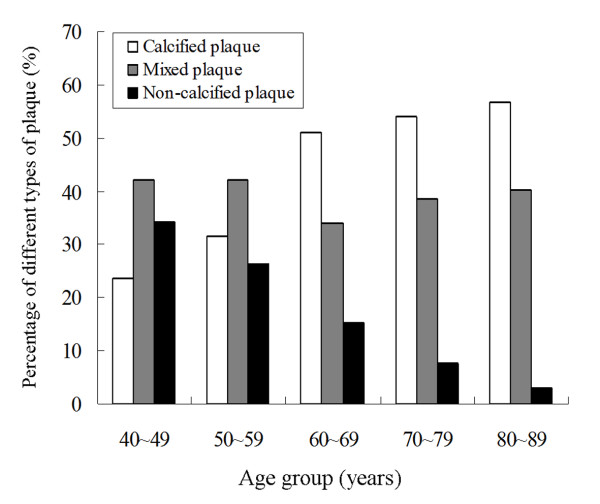
**Graph shows the percentages of different types of plaques in different age groups**. The proportion of calcified plaques increases and that of non-calcified ones decreases as the patients aged.

Different types of plaques and grading of stenosis caused by plaques are shown in Table 2. A total of 480 plaques (4.3 ± 2.9 per patient; range, 1-13) were detected. Calcified plaques (48.8%) were more frequently detected than mixed or non-calcified plaques (*p *< 0.001). Figure 6 shows the percentages of different types of plaques in different age groups. As patients aged, the proportion of calcified plaques increased and that of non-calcified decreased significantly. Furthermore, the calcium score increased as patients aged, with 67.2 ± 110.3, 83.4 ± 185.3, 219.2 ± 319.5, 334.0 ± 621.6 and 584.5 ± 792.5 for age groups 40-49 years, 50-59 years, 60-69 years, 70-79 years and 80-89 years, respectively. Among the different degrees of stenosis, mild narrowing (36.9%) was most common (*p *< 0.001). However, no significant difference between non-obstructive stenosis and obstructive stenosis was observed (50.4% vs. 49.6%, *p *= 0.855).

**Table 2:** The different types of plaque and grading of stenosis in patients

	Value
**Types of plaque**	
Calcified plaque	234 (48.8)
Mixed plaque	183 (38.1)
Non-calcified plaque	63 (13.1)
**Grading of stenosis**	
Normal appearing	65 (13.5)
Mild narrowing	177 (36.9)
Moderate narrowing	119 (24.8)
Severe narrowing	119 (24.8)
**Non-obstructive stenosis**	242 (50.4)
**Obstructive stenosis**	238 (49.6)

Data are expressed as n (%). Non-obstructive stenosis includes normal appearing and mild narrowing. Obstructive stenosis includes moderate and severe narrowing.

### Comparison of CT findings of CAD between different sexes

Comparison of extent of CAD, types of plaques and degrees of stenosis observed on CT between male and female diabetic patients are shown in Table 3. CT findings of CAD between men and women were almost similar in all aspects except that men had more calcified plaques (*p *< 0.05).

**Table 3:** Comparison of coronary artery disease between different genders

	Male (n = 43)	Female (n = 70)	*P*-value
**Extent**			
Diseased vessels	2.7 ± 1.1	2.3 ± 1.1	0.734
Diseased segments	4.5 ± 3.0	3.5 ± 2.5	0.240
**Plaque burden**			
Total plaques	4.6 ± 3.0	3.7 ± 2.7	0.349
Calcified plaque	2.4 ± 2.3	1.5 ± 1.5	0.006
Mixed plaque	1.6 ± 1.9	1.6 ± 1.8	0.954
Non-calcified plaque	0.5 ± 0.9	0.6 ± 0.9	0.606
**Grading of stenosis**			
Normal appearing	0.7 ± 1.2	0.4 ± 0.7	0.056
Mild narrowing	1.7 ± 1.4	1.3 ± 1.0	0.146
Moderate narrowing	1.1 ± 1.2	1.0 ± 1.1	0.582
Severe narrowing	1.1 ± 1.6	1.0 ± 1.7	0.322

Data are expressed as mean ± standard deviation.

## Discussion

This study had four main findings. First, DSCTA could depict coronary plaques and their morphology as well as assess grading of stenosis. Patients did not require excessive preparations to obtain high-quality images. Second, the diabetic patients had a high plaque burden that was mainly distributed in the LAD artery and proximal segment of each coronary vessel. Third, an analysis of plaque composition revealed a relatively high proportion of calcified plaques. Fourth, obstructive stenosis was as prevalent as non-obstructive stenosis. These findings indicated that non-invasive DSCTA was a valuable modality for depicting and evaluating possible coronary atherosclerosis in symptomatic diabetic patients. In addition, results also showed that DM reduced the sex differential in CT findings of CAD.

Three-fourths of the diabetic patients had multi-vessel disease and the plaques involved multiple coronary segments, which indicated that CAD in symptomatic diabetic patients was extensive. This finding was in agreement with those of previous studies [12,13]. The heavy plaque burden in diabetic patients is probably because they have more cardiovascular risk factors resulting from metabolic syndromes [14-16]. In addition, current treatments for DM have limited impact on cardiovascular risk [17]. Multiple coronary plaques in diabetic patients may be related to the increased risk of major adverse cardiac events. It has been established that diabetic patients had a similar risk for cardiac mortality as non-diabetic patients with a history of myocardial infarctions [1].

Our results showed that plaques were more prevalent in the LAD artery and the proximal segment of each vessel in diabetic patients. This finding is similar to those observed in the general population [18-20]. Different susceptibilities of different coronary vessels and segments to atherosclerosis may be explained by their different hemodynamics [21]. However, the precise pathogenetic mechanism still needs further study. Although the plaques in the proximal segments of the vessels may not result in significant stenosis in a short time due to their larger calibre, myocardial ischemia or infarction would be extensive and serious once the lumens were occluded.

Regarding plaque composition, the most frequently detected type in this series was the calcified type followed by the mixed type. This was similar to results of previous studies [13,22,23]. However, one study has shown that non-calcified plaques were the main type of plaques in asymptomatic diabetic patients [24]. In addition, the current study indicated that the proportion of calcified plaques and calcium score increased and that of non-calcified plaques decreased as patients aged. Therefore, the calcium score may underestimate the risk of CAD in diabetic patients, especially in relatively young or asymptomatic individuals.

The future adverse event rate was significantly higher in patients with any coronary plaque than in those with a normal MDCT scan [25]. This may be due to the possibility of each type of plaque causing acute or chronic obstructive stenosis. Non-calcified plaques, which are unstable plaques, were vulnerable and frequently detected in patients with acute coronary artery syndrome [26,27]. Patients with a higher likelihood of stenotic CAD were more likely to have a higher underlying burden of calcified and mixed plaques [28]. Diabetic patients are at a higher risk of CAD: hence, it is important to timely evaluate the potential CAD and treat the remediable plaques.

In this study, the mild narrowing was the most common degree of stenosis, but nearly a half the plaques caused obstructive stenosis in symptomatic patients. This result was consistent with that of a previous study [23]. Obstructive stenosis was seen as a significant indicator of poor prognosis [25]. However, plaques in asymptomatic diabetic patients were usually non-obstructive [24]. The lesion may have been very severe in diabetic patients when symptoms of CAD developed because of the following two reasons. First, the patients may have had DM for many years before it was diagnosed because of lack of typical clinical symptoms [29,30]. Second, painless myocardial ischemia may have developed in a higher percentage of patients and which masked the progress of CAD [31,32]. Therefore, people with risk factors for DM and diabetic patients with cardiovascular risk factors should pay more attention to their blood glucose levels and potential cardiovascular complications.

This study also showed that manifestations of CAD displayed on CT were very similar between men and women. It may be because DM is a major independent cardiovascular risk factor with almost the same risk level in men and women. This result could partly explain the reduced sex differential in CAD mortality and acute CAD risk revealed in previous studies [33,34]. Other studies also showed that the impact of DM on the risk of fatal CAD was significantly greater in women than in men [35,36]. It is believed that DM eliminated the advantage that women had for being at a much lower risk for CAD mortality than men. Therefore, increased attention should be paid to CAD in female diabetic patients.

In light of the severity of CAD in diabetic patients, it is necessary to take measures to prevent or delay its occurrence and development. Diabetic patients should always control their cardiovascular risk factors and recognize the symptoms and signs of potentially fatal CAD as early as possible. Individualized risk estimates and lifestyle advice on physical activity are expected to reduce cardiovascular diseases in high-risk group patients [37]. In contrast, impaired glucose tolerance and type 2 DM should also be suspected in patients with CAD having no previous diagnosis of DM. However, an oral glucose tolerance test was not recommended performing very early after ST-elevation myocardial infarction due to its high false positive rate [38]. As a non-invasive modality, MDCTA has been well established for identification of CAD [5-8]. It is worth mentioning that DSCT not only ensured high-quality images but also promised an impressive reduction in radiation dose [39]. The mean radiation dose for patients who underwent DSCT examination in this study (4.9 mSv) was significantly lower than that in patients who underwent 16-slice (9.8 mSv) or 64-slice (8.6 mSv) MDCT examinations [40].

This study was a cross-sectional study and only diabetic patients with plaques were enrolled. Thus, there was a selection bias. In addition, the patients in this study also had some co-existent cardiovascular risk factors besides type 2 DM, which may affect the results. However, several previous studies had confirmed that the difference in CAD between diabetic and non-diabetic patients was independent of cardiovascular risk factors other than DM [13,22,23]. Thus, the present results demonstrated the current condition of CAD in diabetic patients, which may be more consistent with the practice because diabetic patients often had other concomitant cardiovascular risk factors.

## Conclusion

Coronary CTA detected a high plaque burden in symptomatic patients with type 2 DM. The plaques, which were mainly calcified, were more frequently detected in the proximal segment of the LAD artery. Increased attention should be paid to the obstructive stenosis, which had a similar prevalence to non-obstructive stenosis. In addition, DM reduced the sex differential in the CT findings of CAD. Thus, DSCT can be used to detect potential CAD in symptomatic diabetic patients and provide additional information for evaluating its severity and managing treatment.

## Competing interests

The authors declare that they have no competing interests.

## Authors' contributions

All authors participated in the design and coordination of the study, reviewed the analysis and took part in writing the manuscript. They also read and approved the final manuscript.
